# Metabolic reprogramming induced by DCA enhances cisplatin sensitivity through increasing mitochondrial oxidative stress in cholangiocarcinoma

**DOI:** 10.3389/fphar.2023.1128312

**Published:** 2023-09-25

**Authors:** Hanjiao Qin, Ge Zheng, Qiao Li, Luyan Shen

**Affiliations:** ^1^ Department of Radiation Oncology, The Second Hospital of Jilin University, Changchun, China; ^2^ Department of Hepatobiliary and Pancreatic Surgery, Second Hospital of Jilin University, Changchun, China; ^3^ Second Hospital of Jilin University, Changchun, China; ^4^ Key Laboratory of Pathobiology, Department of Pathophysiology, Ministry of Education, Jilin University, Changchun, China

**Keywords:** metabolic reprogramming, redox homeostasis, autophagy, DCA, cisplatin sensitivity, cholangiocarcinoma

## Abstract

**Background:** Cholangiocarcinoma has obvious primary multidrug resistance and is generally resistant to cisplatin and other chemotherapy drugs and high glycolytic levels may be associated with chemotherapy resistance of cholangiocarcinoma cells. Dichloroacetate (DCA) is a specific inhibitor of PDK, which can promote mitochondrial aerobic oxidation process by activating PDH. In the past few years, there have been an increasing number of studies supporting the action of DCA against cancer, which also provided evidence for targeting metabolism to enhance the efficacy of cholangiocarcinoma chemotherapy.

**Methods:** Glucose uptake and lactic acid secretion were used to detect cell metabolism level. Cell apoptosis and cell cycle were detected to confirm cell fate induced by cisplatin combined with DCA. Mito-TEMPO was used to inhibit mtROS to explore the relationship between oxidative stress and cell cycle arrest induced by DCA under cisplatin stress. Finally, PCR array and autophagy inhibitor CQ were used to explore the potential protective mechanism under cell stress.

**Results:** DCA changed the metabolic model from glycolysis to aerobic oxidation in cholangiocarcinoma cells under cisplatin stress. This metabolic reprogramming increased mitochondrial reactive oxygen species (mtROS) levels, which promoted cell cycle arrest, increased the expression of antioxidant genes and activated autophagy. Inhibition of autophagy further increased the synergistic effect of DCA and cisplatin.

**Conclusion:** DCA increased cisplatin sensitivity in cholangiocarcinoma cells via increasing the mitochondria oxidative stress and cell growth inhibition. Synergistic effects of DCA and CQ were observed in cholangiocarcinoma cells, which further increased the cisplatin sensitivity via both metabolic reprogramming and inhibition of the stress response autophagy.

## 1 Introduction

Cholangiocarcinoma (CCA) is an epithelial tumor of the bile duct, which shows typical histological features of differentiation of bile duct cells (Rizvi and Gores, 2013). Considering that up to 85% of CCA patients experience recurrence within 3 years after resection, better adjuvant therapy strategies are needed (Takada et al., 2002; Tamandl et al., 2008). Many studies have indicated that CCA has obvious primary multidrug resistance and is generally insensitive to cisplatin and other chemotherapy drugs (Zheng et al., 2022). Recent studies have shown that the high level of glycolysis pathway proteins in CCA met its proliferation requirements, providing favorable conditions for tumorigenesis and development (Pant et al., 2020). Yokoi et al. found that inhibition of the Akt/mTOR (mammalian target of rapamycin complex) pathway—which initiates aerobic glycolysis and lactic acid assembly—could enhance the sensitivity of CCA cells to chemotherapy (Yokoi et al., 2018), suggesting that targeting CCA cell metabolism may provide a possibility for improving the chemotherapeutic efficacy.

Some progress has been made in the study of glucose metabolism reprogramming in CCA. Through bioinformatics analysis, some scholars have found that abnormal metabolism promoted the proliferation of CCA cells. Furthermore, Seahorse XF96 Extracellular Flux Analyzer analysis showed that metformin could promote aerobic oxidation, inhibit the Warburg effect of CCA cells, and reduce the expression of lactate dehydrogenase A (Tang et al., 2018), thereby playing a part in anti-proliferation and anti-metastasis effects in CCA cells. It has been shown that changes in glucose metabolism can be observed in SIRT3-knockout mice, suggesting that a low expression level of SIRT3 correlates with a high glycolysis level in CCA. SIRT3 prevents the Warburg effect in CCA cells and xenograft mouse models by inhibiting the HIF1α/PDK1 pathway (Xu et al., 2019). These studies suggest that reprogramming the metabolic model of CCA may be a potential target to inhibit the development of CCA and improve the efficiency of chemotherapy.

As a specific inhibitor of pyruvate dehydrogenase kinase (PDK), DCA can enhance mitochondrial aerobic oxidation by activating pyruvate dehydrogenase (PDH). DCA has been used to treat type II diabetes (Mayers et al., 2005), congenital mitochondrial diseases (Michelakis et al., 2008), and other metabolic diseases on the basis of reducing lactic acid levels. Considering the great interest in the abnormal metabolism of tumors and its influence on tumorigenesis, development, and treatment of tumors, DCA has been used in a variety of antitumor therapy studies [oral carcinoma (Ruggieri et al., 2015), lung cancer (Bonnet et al., 2007), endometrial cancer, and breast cancer (Michelakis et al., 2008)]. Overexpression of PDK3 in tumor cells (HeLa, IMR32, and colo320DM cells) can increase metabolic conversion to glycolysis and increase the risk of drug resistance and tumor recurrence (Lu et al., 2008). In colorectal cancer cells, inhibition of PDK1–4 by DCA increased the activity of PDH and promoted the catalysis of pyruvate into acetyl-CoA for oxidative phosphorylation, which led to cell cycle arrest and cell apoptosis (Madhok et al., 2010). Thus, given that hyperactive glycolytic pathways in tumor cells lead to chemotherapy resistance, targeting metabolism with DCA can be a potential strategy for synergistic chemotherapy.

DCA-enhanced chemotherapy is a potential option, but the role of DCA in chemotherapy resistance remains unclear. Previous studies have shown that DCA can increase the levels of mitochondrial reactive oxygen species (mtROS) by changing cell metabolism (Sun et al., 2011; Niewisch et al., 2012). As mitochondria are the ultimate location of catabolism of three major nutrients and energy generation, mitochondrial function regulates the redox state of the cells and is also the target of redox signaling response and damage. ROS are a ubiquitous by-product of all cellular metabolic processes. The multiple downstream effects of ROS and their action on cell fate depend on their intracellular concentration (Cairns et al., 2011). Low levels of ROS provide a beneficial effect as a second intracellular messenger supporting cell proliferation and survival pathways (Giannoni et al., 2005). However, when ROS levels are extremely elevated, they can cause harmful oxidative stress, which leads to cell death (Fruehauf and Meyskens, 2007). Therefore, exploring the role of ROS in chemotherapy resistance may provide a theoretical basis for improving efficacy by targeting metabolism.

In this study, we detected the glucose uptake and lactic acid secretion to explore cell metabolism level. Flow cytometry, RT-qPCR and western blot were used to detect cell apoptosis, flow cytometry, RT-qPCR and cell population doubling time to detect cell cycle. Intracellular and mitochondrial ROS levels were detected by fluorescent dye staining, and Mito-TEMPO was used to inhibit mtROS to explore the relationship between oxidative stress and cell cycle arrest. Finally, autophagy inhibitor CQ was used to explore the potential protective mechanism under cell stress. To explore the role of metabolic reprogramming in cisplatin resistance in CCA cells may further clarify the role of oxidative stress signaling mediated by metabolic dysfunction in cell fate and provide evidence for effective metabolic-targeted treatment program on elevating cancer chemosensitivity.

## 2 Materials and methods

### 2.1 Reagents and antibodies

Cisplatin, DCA, chloroquine diphosphate salt (CQ), and 3-(4,5-dimetrylthiazol-2-yl)-2,5-diphenyltetrazolium bromide (MTT) were purchased from Sigma-Aldrich (St. Louis, MO, United States). Next, 2′,7′-dichlorofluorescin diacetate (DCFH-DA) was purchased from Beyotime Institute of Biotechnology (Shanghai, China). MitoSOX™ Red (mitochondrial superoxide indicator) and enhanced chemiluminescence (ECL) reagents were purchased from Thermo Scientific (Rockford, IL, United States). Mito-TEMPO (mitochondria-targeted superoxide dismutase mimetic) was purchased from Selleck (Houston, TX, United States). The following antibodies were used: anti-β-actin (60008-1-Ig), anti-p62 (18420-1-AP), anti-LC3B (18725-1-AP), anti-Sirt3 (10099-1-AP), anti-SOD2 (24127-1-AP), peroxidase-conjugated AffiniPure goat anti-mouse IgG (H+L) (SA00001-1), and peroxidase-conjugated AffiniPure goat anti-rabbit IgG (H+L) (SA00001-2) (Proteintech, Chicago, IL, United States).

### 2.2 Cell culture

The human CCA cell line QBC939 was obtained from the Third Military Medical University and human CCA cell line RBE was obtained from the Cell Bank of the Institute of Biochemistry and Cell Biology (Shanghai, China). The cell line was maintained at 37°C in a 5% CO_2_ and 95% air atmosphere in Roswell Park Memorial Institute-1640 culture medium (Gibco Life Technologies, Carlsbad, CA, United States) supplemented with 10% fetal bovine serum (Invitrogen, Carlsbad, CA, United States), 100 U/mL penicillin, and 100 U/mL streptomycin.

### 2.3 Cellular viability assays

Cellular viability was measured with MTT assays. Cells were seeded in 96-well plates at a density of 1 × 10^4^ cells/well. After the cells were exposed to cisplatin, 20 μL MTT solution (5 mg/mL) was added to each well and the cells were incubated for 4 h. DMSO (Beijing Chemical Industry Co., Ltd., Beijing, China) was then added to the wells to solubilize the formazan products after elimination of the media. Absorbance was recorded at 570 nm using a CLARIOstar microplate reader (BMG Labtech, Offenburg, Germany). The growth inhibition rate was calculated as follows: Inhibition (%) = [1—(absorbance of the experimental group/absorbance of the control group)] × 100.

### 2.4 Glucose and lactate concentration measurement

The cells were seeded in six-well plates at 5 × 10^5^ cells/well. Following overnight incubation at 37°C, the medium was changed to fresh complete medium. After 24 h, the medium was collected, after which the proteins were extracted through sonication and quantified using a Bradford Protein Assay kit (Beyotime Institute of Biotechnology). Then, glucose and lactate concentrations were determined using glucose (RsBio, Shanghai, China) and lactate assay kits (Jiancheng Bio, Nanjing, China), respectively. Glucose consumption in each group was calculated as follows: Glucose consumption = glucose concentration (fresh complete medium)—glucose concentration (experimental group), normalized to the protein content.

### 2.5 Flow cytometry analysis

Annexin V-FITC (Annexin V Apoptosis Detection Kit II, BD Biosciences, San Diego, CA, United States) was used to detect cell apoptosis and propidium iodide (PI) was used to detect cell cycle. Exponentially growing QBC939 cells were seeded in 6 well culture plates at a density of 2 × 10^5^ cells/well. After exposure to different experimental conditions, cells were trypsinized and resuspended in 1640 medium with 10% FBS at a concentration of 1 × 10^6^ cells/ml. Samples were examined using the BD Accuri™ C6 Plus personal flow cytometer (Becton, Dickinson and Company, Franklin Lakes, NJ, United States).

### 2.6 Immunofluorescence staining and and fluorescence microscopy

The intracellular reactive oxygen species (ROS) and intramitochondrial superoxide anion (O^2−^) were determined using DCFH-DA and MitoSOX™ Red, respectively. Cells were seeded onto coverslips in 24-well plates (5 × 10^4^ cells/well) overnight and exposured to different experimental conditions. After incubation with DCFH-DA (10 μM) or MitoSOX Red (5 μM) at 37°C and 5% CO_2_ for 20 min, cells were washed with cold PBS three times. After mounting, the images were acquired by an Echo Lab Revolve microscope (San Diego, CA, United States).

### 2.7 Western blot analysis

Cells subjected to desired treatments were harvested, washed twice with cold PBS, and then gently scraped into 120 μL of RIPA buffer. Cell lysates were sonicated for 30 s on ice and then lysed at 4°C for 45 min. Cell lysates were centrifuged at 3,000 × g for 15 min, and supernatant protein concentrations were determined using the Bio-Rad kit (Pierce Biotechnology, Inc., Rockford, IL, United States). For western blot analysis, equivalent amounts of lysate proteins (30–50 μg) were separated by 12% w/v SDS-polyacrylamide gel electrophoresis and transferred onto immobilon-P transfer membranes (Millipore Corp., Bedford, MA, United States of America). Membranes were blocked with 5% (w/v) skim milk in buffer [PBST: 10 mM Tris-HCl (pH 7.6), 100 mM NaCl and 0.1% (v/v) Tween-20] for 1 h at room temperature, then incubated with the desired primary antibody overnight at 4°C. The following day, membranes were washed with PBST and incubated with horseradish peroxidase-conjugated secondary antibodies (1:2000; Proteintech, Chicago, IL) for 1 h at room temperature. After washing the membranes with PBST, immunodetection was performed using ECL reagent (Thermo Fisher Scientific) and visualized using a Syngene Bio Imaging (Synoptics, Cambridge, UK). Protein levels were quantified by densitometry using Quantity One software (Bio-Rad Laboratories, Inc., Hercules, CA, United States), normalized to β-actin.

### 2.8 Cell cycle analysis

DNA Content Quantitation Assay (Cell Cycle) (Solarbio life sciences, Beijing, China) was used to detect cell cycle. Exponentially growing QBC939 cells were seeded in 6 well culture plates at a density of 2 × 10^5^ cells/well. After exposure to different experimental conditions, cells were harvested with trypsin and the cell pellets were fixed in ice-cold 70% ethanol at 4°C overnight. Then the fixed cells were recovered by centrifugation, rewashed with PBS, incubated with 100 μL RNase at 37°C for 30 min, and stained with 400 μL PI at 4°C for 30 min in the dark prior. Samples were examined using the BD Accuri™ C6 Plus personal flow cytometer (Becton, Dickinson and Company, Franklin Lakes, NJ, United States).

### 2.9 Human autophagy RT^2^ profiler polymerase chain reaction (PCR) array

The expression levels of 84 key genes in autophagy were determined by RT^2^ Profiler™ PCR Array Human Autophagy (SABiosciences-Qiagen, Hilden, Germany). Total RNA was isolated from cultured cells, and 1 μg of total RNA was reverse-transcribed to singlestranded cDNA using the RT2 First Strand kit (SABiosciences-Qiagen, Hilden, Germany). The expression levels of genes of interest were determined by quantitative PCR using CFX96 Touch™ Real-Time PCR Detection System (Bio Rad Laboratories, Inc., Hercules, CA, United States) with SYBR Green fluorophore using the RT^2^ SYBR Green Master Mix (SABiosciences-Qiagen, Hilden, Germany). The reaction program involved 40 cycles of 95°C for 10 min, 95°C for 15 s and 60°C for 1 min. The results were analyzed using the manufacturer’s software and relative gene expression was quantified using the 2^−ΔΔCq^ method (Livak and Schmittgen, 2001). The altered expression of the 84 genes was displayed using heat imaging with normalization to ACTB, B2M and GAPDH.

### 2.10 Relative reverse transcription-quantitative polymerase chain reaction (RT-qPCR)

Total cellular RNA was extracted using TRIzol™ reagent (Invitrogen) and reverse transcription was performed to generate cDNA, which was then amplified by quantitative real-time PCR (RT-qPCR). The sequences of the primers used are listed in Table 1. RT-qPCR was performed using a 2× SYBR Green qPCR Master Mix (B21202, Bimake, China) and the following conditions: 95.0°C for 30 s–10 min, 40 cycles of 95.0°C for 15 s and 60.0°C for 30–60 s. A melting curve was detected from 60°C to 95°C to confirm the desired PCR product. Each sample was analyzed in triplicate in the CFX96 Touch™ Real-Time PCR Detection System (Bio-Rad Laboratories, Inc., Hercules, CA, United States). The relative expression was calculated by ΔCt among different experimental groups normalized to ACTB (B661102, Sangon Biotech, China) expression.

**Table 1:** Primers for RT-qPCR analysis.

Gene name	Primer sequences
GAPDH	F: 5′-GGA​GCG​AGA​TCC​CTC​CAA​AAT-3′
	R: 5′-GGC​TGT​TGT​CAT​ACT​TCT​CAT​GG-3′
BAX	F: 5′-CCC​GAG​AGG​TCT​TTT​TCC​GAG-3′
	R: 5′-CCA​GCC​CAT​GAT​GGT​TCT​GAT-3′
BAK1	F: 5′-ATG​GTC​ACC​TTA​CCT​CTG​CAA-3′
	R: 5′-TCA​TAG​CGT​CGG​TTG​ATG​TCG-3′
BCL2	F: 5′-GGT​GGG​GTC​ATG​TGT​GTG-3′
	R: 5′-CGG​TTC​AGG​TAC​TCA​GTC​ATC​C-3′
BCL2L1	F: 5′-GAG​CTG​GTG​GTT​GAC​TTT​CTC-3′
	R: 5′-TCC​ATC​TCC​GAT​TCA​GTC​CCT-3′
MCL1	F: 5′-GTG​CCT​TTG​TGG​CTA​AAC​ACT-3′
	R: 5′-AGT​CCC​GTT​TTG​TCC​TTA​CGA-3′
CDKN1A	F: 5′-TGT​CCG​TCA​GAA​CCC​ATG​C-3′
	R: 5′-AAA​GTC​GAA​GTT​CCA​TCG​CTC-3′
CDKN2A	F: 5′-GGG​TTT​TCG​TGG​TTC​ACA​TCC-3′
	R: 5′-CTA​GAC​GCT​GGC​TCC​TCA​GTA-3′
CDKN1B	F: 5′-ATC​ACA​AAC​CCC​TAG​AGG​GCA-3′
	R: 5′-GGG​TCT​GTA​GTA​GAA​CTC​GGG-3′
ATG5	F: 5′-AGA​AGC​TGT​TTC​GTC​CTG​TGG-3′
	R: 5′-AGG​TGT​TTC​CAA​CAT​TGG​CTC-3′
ATG7	F: 5′-ATG​ATC​CCT​GTA​ACT​TAG​CCC​A-3′
	R: 5′-CAC​GGA​AGC​AAA​CAA​CTT​CAA​C-3′
ATG12	F: 5′-CTG​CTG​GCG​ACA​CCA​AGA​AA-3′
	R: 5′-CGT​GTT​CGC​TCT​ACT​GCC​C-3′
MAP1LC3A	F: 5′-AAC​ATG​AGC​GAG​TTG​GTC​AAG-3′
	R: 5′-GCT​CGT​AGA​TGT​CCG​CGA​T-3′

F, forward; R, reverse; BAX, BCL2-associated X; BAK1, BCL2 antagonist/killer 1; BCL2, B-cell lymphoma 2; BCL2L1, BCL2 like 1; MCL1, myeloid cell leukemia sequence 1; CDKN2A, cyclin-dependent kinase inhibitor 2A; CDKN1A, cyclin-dependent kinase inhibitor 1A; CDKN1B, cyclin-dependent kinase inhibitor 1B; ATG5, autophagy-related 5; ATG7, autophagy-related 7; ATG12, autophagy-related 12; and MAP1LC3A, microtubule-associated protein 1 light chain 3 alpha.

### 2.11 Statistical analysis

Data are representative of three independent experiments, each performed in triplicate. Statistical analysis of the data was performed using a one way analysis of variance with IBM SPSS version 22.0 (IBM SPSS, Armonk, NY, United States). Tukey’s *post hoc* test was used to determine the significance for all pairwise comparisons of interest. *p* < 0.05 was considered to indicate a statistically significant difference.

## 3 Results

### 3.1 DCA-mediated metabolic reprogramming increases the sensitivity of CCA cells to cisplatin

**FIGURE 1:**
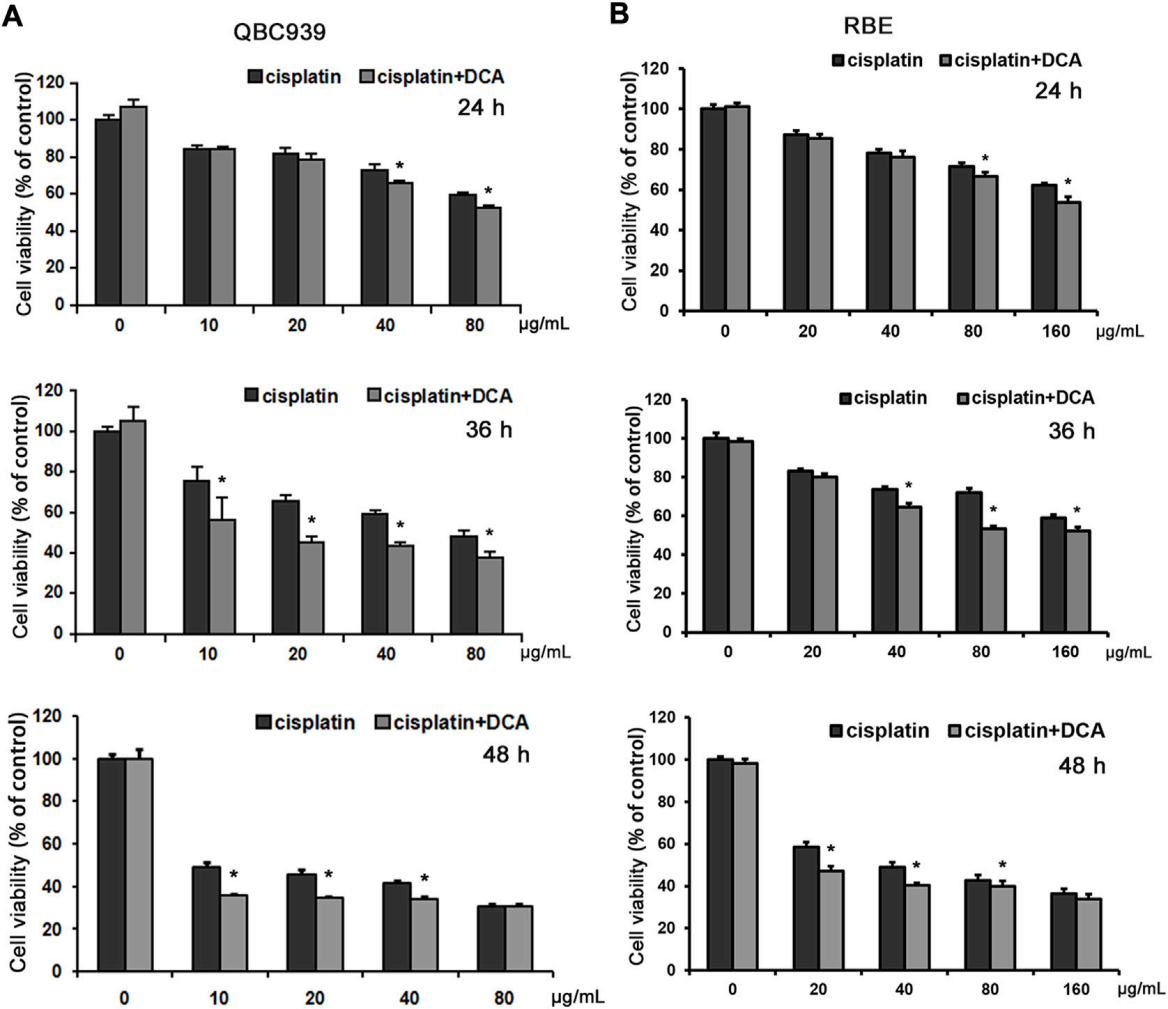
DCA increases the sensitivity of QBC939 and RBE cells to cisplatin. **(A)** QBC939 cells were treated with varying doses of cisplatin combined with DCA (20 mM) for 24 h, 36 h and 48 h **(B)** RBE cells were treated with varying doses of cisplatin combined with DCA (20 mM) for 24 h, 36 h and 48 h. Cell viability was determined using MTT assay (mean ± SD, *n* = 3; *p < 0.05, vs. cisplatin).

Previous studies have shown that CCA QBC939 cells are more resistant to cisplatin and that they are more inclined to the glycolytic pathway to resist extracellular stress (Qu et al., 2017), suggesting that cisplatin resistance can be reversed through cell metabolic reprogramming. Thus, we used a PDK inhibitor DCA to transform the metabolic model of QBC939 cells by activating PDH for follow-up experiments. QBC939 cells were treated with varying doses of cisplatin combined with DCA (20 mM) for 24, 36, and 48 h, and the cell viability was detected using MTT assay. As shown in Figure 1A, compared with the groups treated with cisplatin, the cell viability of QBC939 cells was significantly reduced in the groups treated with cisplatin combined with DCA. Meanwhile, CCA RBE cells were treated with varying doses of cisplatin combined with DCA (20 mM) for 24, 36, and 48 h to detected cell viability using MTT assay. Consistent with the results of QBC939 cells, the cell viability of RBE cells was reduced in the groups treated with cisplatin combined with DCA (Figure 1B).

**FIGURE 2:**
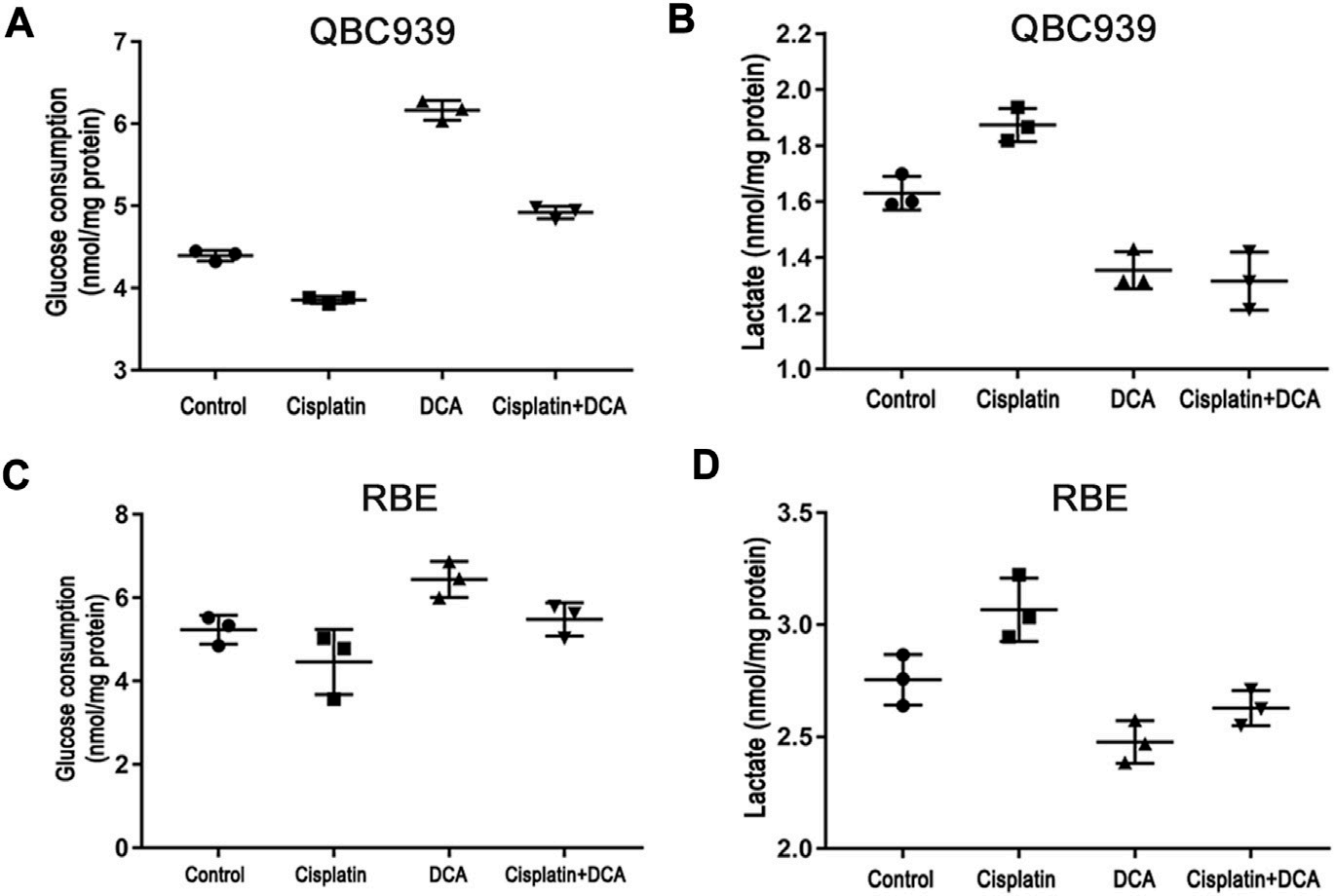
DCA induces metabolism transformation in QBC939 and RBE cells. **(A)** Glucose consumption and **(B)** lactate production were measured in the culture media from QBC939 cells using glucose and lactate kits and were normalized to the protein content. **(C)** Glucose consumption and **(D)** lactate production were measured in the culture media from RBE cells using glucose and lactate kits and were normalized to the protein content. (mean ± SD, *n* = 3).

We also used the glucose and lactate assay kits to analyze the changes in glucose uptake and lactate secretion in QBC939 cells and RBE cells treated with cisplatin with or without DCA. The results in Figures 2A, B showed that the level of glucose uptake significantly increased and the level of lactic acid secretion decreased in the DCA groups in QBC939 cells, indicating that the cellular metabolism of QBC939 cells shifted from glycolysis to aerobic oxidation. In contrast, the level of glucose uptake decreased and the level of lactic acid secretion increased in QBC939 cells after the treatment with cisplatin only. These changes were reversed in the groups treated with cisplatin combined with DCA, that is, the level of glucose uptake increased and the level of lactic acid secretion decreased compared with the cisplatin groups. The results in Figures 2C, D showed that DCA increased the level of glucose uptake induced by cisplatin and decreased the level of lactic acid secretion induced by cisplatin, but, the effect of DCA in RBE cells was weaker than that in QBC939 cells. These results suggest that cisplatin sensitivity increased by DCA is related to DCA-mediated metabolic reprogramming.

### 3.2 DCA-induced oxidative stress enhances cisplatin cytotoxicity by cell cycle arrest and not cell apoptosis in QBC939 cells

**FIGURE 3:**
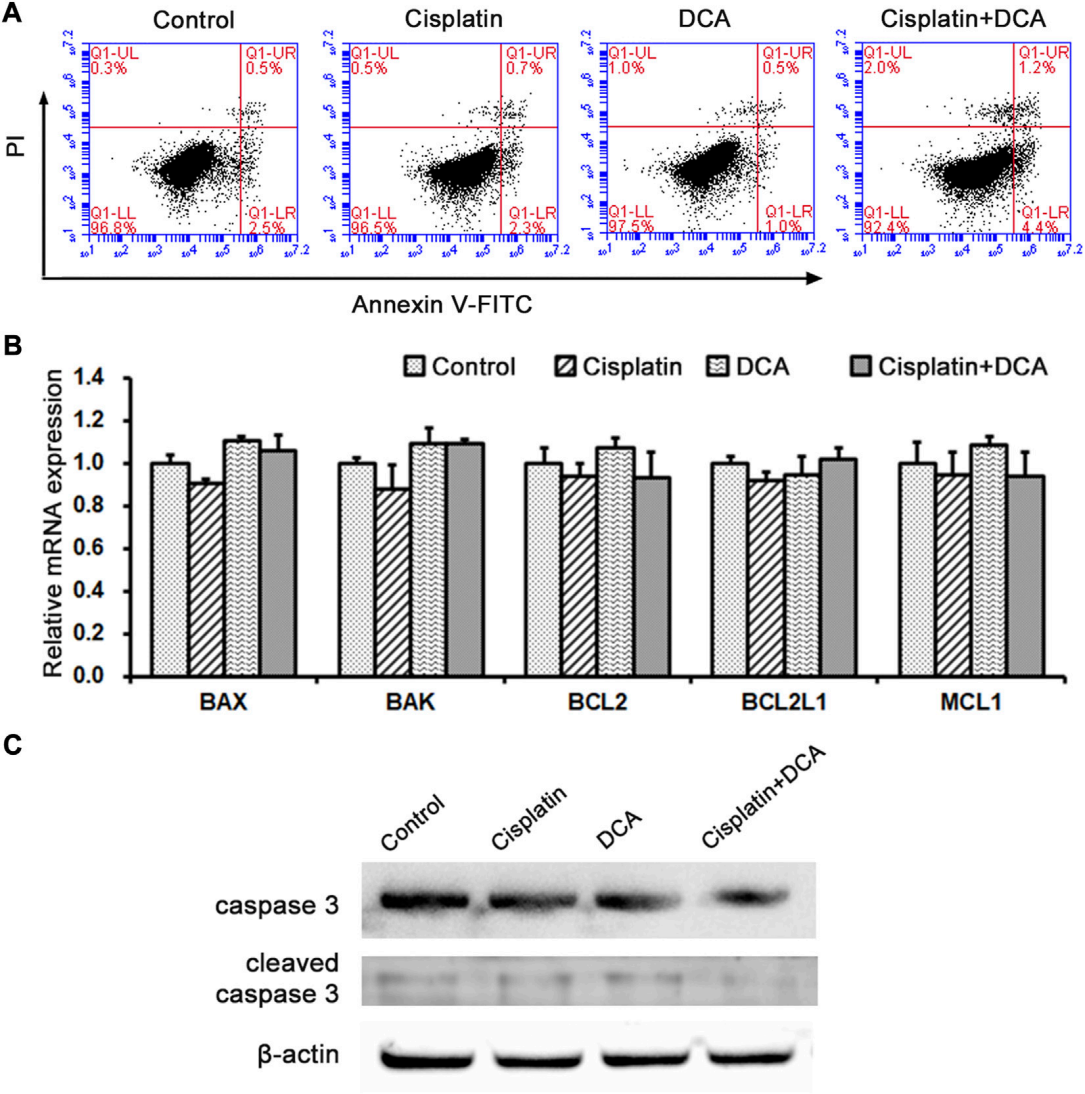
Metabolism transformation induced by DCA does not activate cell apoptosis in QBC939 cells. **(A)** Cell apoptosis was assessed by staining with Annexin V-FITC and PI in QBC939 cells. **(B)** RT-qPCR detection of apoptosis-associated genes in QBC939 cells. **(C)** Western blot detection of caspase-3 protein in QBC939 cells. (mean ± SD, *n* = 3).

In order to further explore the mechanism of DCA enhanced cisplatin efficacy by metabolic reprogramming, we chose QBC939 cells for following experiments. Based on the MTT results, Annexin V-FITC was used to detect the ratio of cell apoptosis in QBC939 cells treated with cisplatin with or without DCA by flow cytometry. As shown in Figure 3A, there was no significant difference in apoptosis cell ratio between different treatment groups. We also used RT-qPCR to detect the expression of Bcl-2 family apoptosis-related genes in QBC939 cells treated with cisplatin with or without DCA (Figure 3B). We found no significant changes in the gene expression of BAX, BAK, BCL2, BCL2L1, and MCL1. And the expression of cleaved caspase 3 detected by western blot had no significant changes (Figure 3C), demonstrating that DCA did not activate cell apoptosis in QBC939 cells.

**FIGURE 4:**
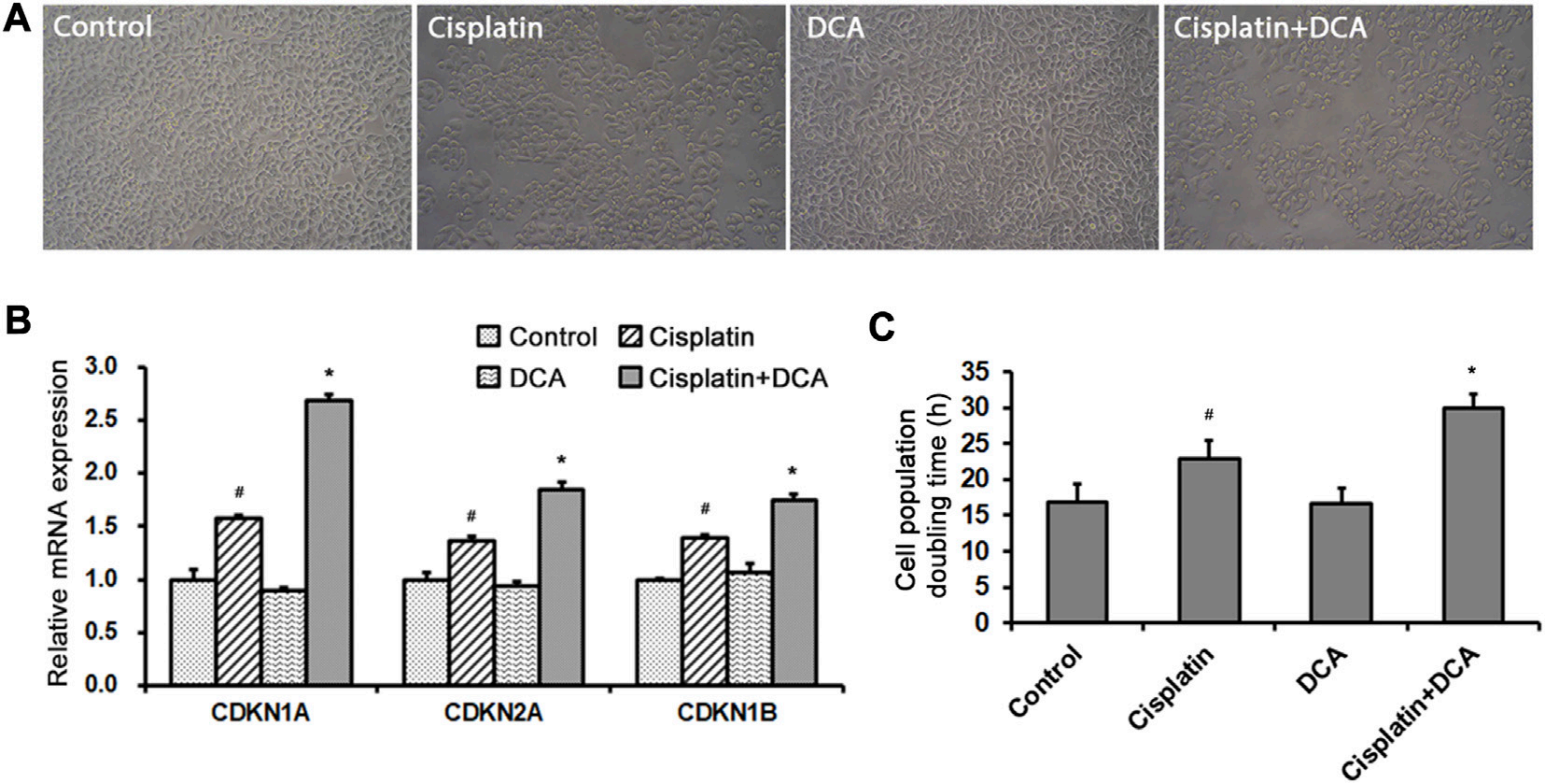
DCA enhances cisplatin-induced cell cycle arrest. **(A)** QBC939 cells were treated with cisplatin (10 μg/mL) with or without DCA (20 mM) for 36 h. Cell morphology was observed using an inverted phase-contrast microscope at ×200 magnification. **(B)** RT-qPCR detection of cell cycle–associated genes in QBC939 cells (mean ± SD, n = 3; ^#^ *p* < 0.05, vs. control, and *p < 0.05, vs. cisplatin). **(C)** QBC939 cells were treated with cisplatin (10 μg/mL) with or without DCA (20 mM) for 24 h. Cell population doubling time was calculated by cell count (mean ± SD, *n* = 3; ^#^ *p* < 0.05, vs. control, and *p < 0.05, vs. cisplatin).

Optical microscopy was used to examine cellular morphological changes. Compared with the controls, the total cell count decreased and the cells became round after the treatment with cisplatin. The total cell count and the number of round cells were lower in the groups treated with cisplatin combined with DCA than in the groups treated with cisplatin only, and no fragmented cells were observed (Figure 4A). RT-qPCR was used to detect the expression of cell cycle–related genes CDKN1A, CDKN2A, and CDKN1B in QBC939 cells treated with cisplatin with or without DCA. As shown in Figure 4B, the expression of CDKN1A, CDKN2A, and CDKN1B genes increased in the groups treated with cisplatin, and the expression of these genes significantly increased in the groups treated with cisplatin combined with DCA compared with the groups treated with cisplatin only, indicating that cell cycle arrest occurred. Then, cell counting was used to calculate the cell population doubling time. As shown in Figure 4C, the cell population doubling time increased from 17 h in the control groups to 23 h in the cisplatin groups and even 30 h in the groups treated with cisplatin combined with DCA.

**FIGURE 5:**
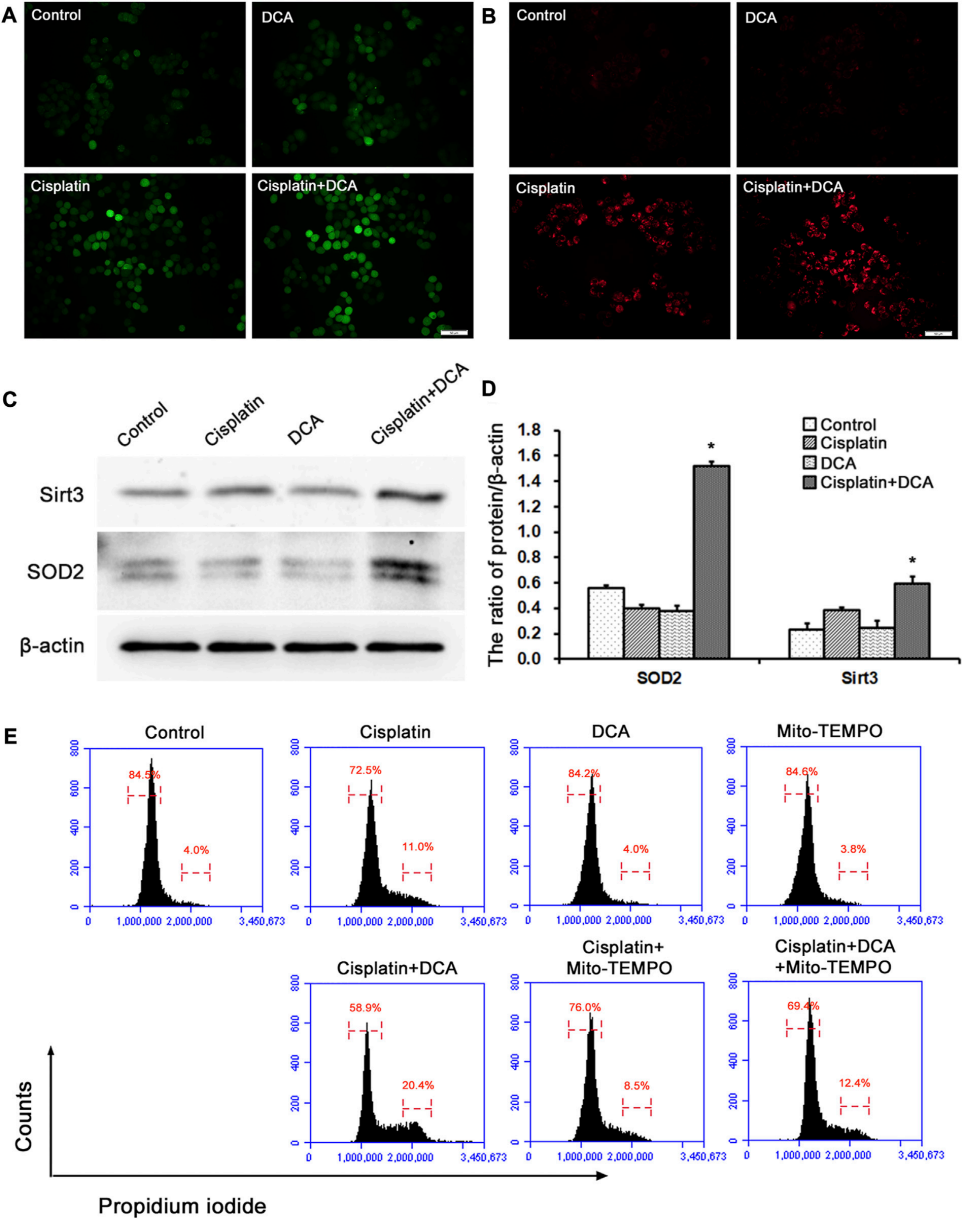
DCA increases the ROS level induced by cisplatin and activates the antioxidant system in QBC939 cells. **(A)** The intracellular ROS staining with DCFH-DA was observed with fluorescence microscopy in QBC939 cells (scale bar, 50 μm). **(B)** The intramitochondrial superoxide anion staining with MitoSOX™ Red was observed with fluorescence microscopy in QBC939 cells (scale bar, 50 μm). **(C)** Western blot detection of antioxidant proteins in QBC939 cells. **(D)** Quantitation of Sirt3 and SOD2 protein levels (mean ± SD, n = 3; *p < 0.05, vs. cisplatin). **(E)** Flow cytometric analysis of QBC939 cells staining with propidium iodide.

To explore the cause of cell cycle arrest induced by DCA, intracellular ROS were stained with fluorescent dye DCFH-DA to assess the redox balance in QBC939 cells. The results (Figure 5A) showed that the green fluorescence intensity increased in the groups treated with cisplatin, and the green fluorescence intensity was significantly higher in the groups treated with cisplatin combined with DCA than in the groups treated with cisplatin only, indicating that the level of intracellular ROS increased. Meanwhile, we also stained intramitochondrial superoxide anion with the fluorescent dye MitoSOX™ Red to detect mtROS level using fluorescence microscopy. Consistent with the results shown in Figure 5A, the red fluorescence intensity increased in the groups treated with cisplatin, and the red fluorescence intensity was significantly higher in the groups treated with cisplatin combined with DCA than in the groups treated with cisplatin only (Figure 5B). These findings suggested that DCA-induced metabolic shift from glycolysis to aerobic oxidation activated mitochondrial oxidative stress, which may be related to cell cycle arrest.

Next, we examined the protein expression levels of mitochondrial antioxidant-related signaling molecules by western blot. We found that the protein expression levels of Sirt3 and SOD2 were higher in the groups treated with cisplatin combined with DCA than in the control and cisplatin groups, and there were no significant increases in these proteins’ expression levels in the cisplatin groups compared with the control groups (Figures 5C, D). To confirm the relationship between mtROS and cell cycle arrest induced by DCA, we used Mito-TEMPO to scavenge mtROS and detected cell cycle by flow cytometry. As showed in Figure 5E, cell cycle arrest occurred in QBC939 cells treated with cisplatin, and cell cycle arrest was more severe when combined treatment with DCA. Cell cycle arrest was relieved after treatment with mito-TEMPO to inhibit the increased mtROS induced by DCA. These results suggested that cisplatin combined with DCA destroyed the redox homeostasis in QBC939 cells, which retrogradely activated the cell cycle arrest machinery and antioxidant system to promote the expression of related molecules.

### 3.3 Combinational use of DCA with autophagic inhibitor CQ enchanced the cisplatin sensitivity in QBC939 cells

Our results demonstrated that treatment with cisplatin combined with DCA enhanced the growth-inhibition effect of cisplatin in QBC939 cells by transforming the metabolic model and activating mitochondrial oxidative stress, but the enhancing effect was only approximately 20%, suggesting that there may be a cell protective mechanism against cisplatin cytotoxicity. Previous studies have shown that mtROS could activate autophagy (Chen et al., 2016; Yuan et al., 2018) and autophagy was activated as a protective mechanism in CCA cells in response to cellular stress (Qu et al., 2017). Thus, we further explored the action of DCA-induced metabolic reprogramming in cisplatin cytotoxicity to CCA cells from the perspective of autophagy.

**FIGURE 6:**
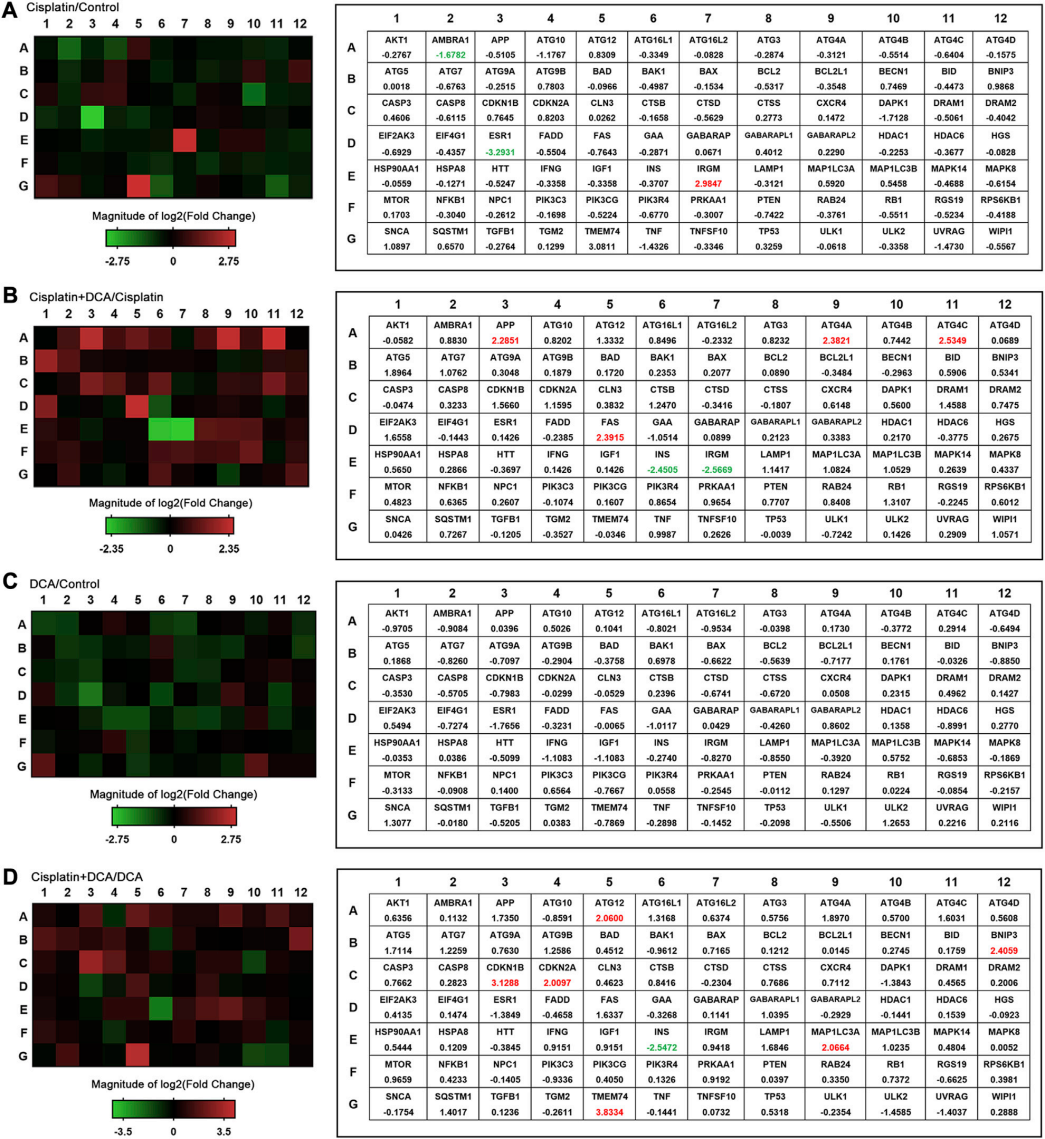
Autophagy pathway is altered in QBC939 cells. The expression of autophagy-related genes (84 genes) was evaluated in cells using a human autophagy PCR array. QBC939 cells were treated with 10 μg/mL cisplatin, 20 mM DCA, or 10 μg/mL cisplatin combined with DCA (20 mM) for 12 h. The changes in gene expression are indicated in the heat map, that is **(A)** cisplatin vs. control, **(B)** cisplatin + DCA vs. cisplatin, **(C)** DCA vs. control, and **(D)** cisplatin + DCA vs. DCA. Red indicates upregulation, and green indicates downregulation.

To comprehensively evaluate intracellular autophagy levels, we used a human autophagy RT^2^ Profiler PCR array to detect the expression of 84 autophagy-associated genes in QBC939 cells treated with cisplatin, DCA, and cisplatin combined with DCA. As shown in Figures 6A, C, the expression of autophagy-related genes was not significantly increased in the cisplatin groups compared with the control groups; consistent with these results, the expression of autophagy genes was not significantly increased in the DCA groups compared with the control groups. However, the expression of autophagy genes was significantly increased in the groups treated with cisplatin combined with DCA compared with the groups treated with cisplatin only and DCA only (Figures 6B, D).

**FIGURE 7:**
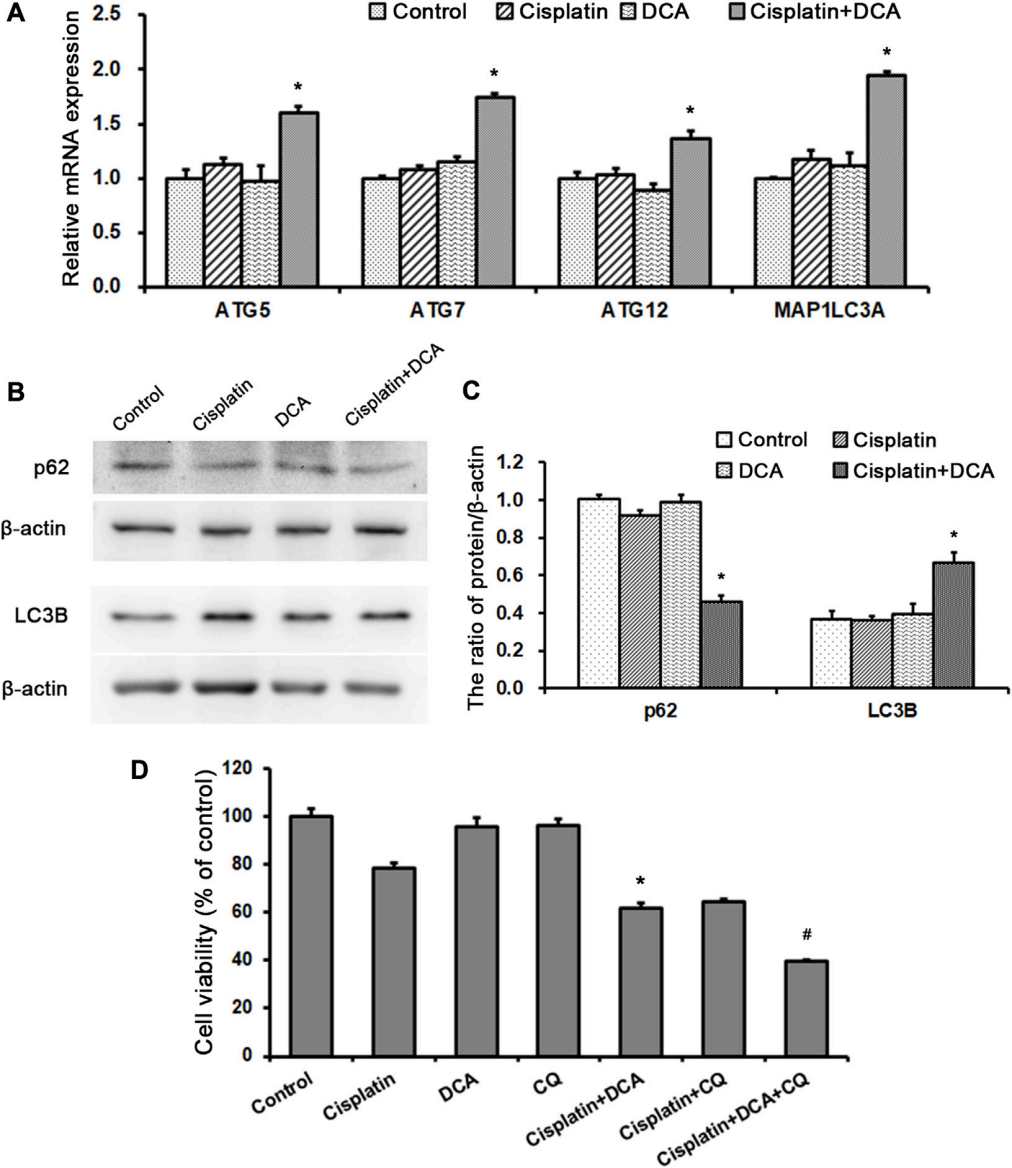
DCA activates autophagy to resist cisplatin toxicity in QBC939 cells. **(A)** RT-qPCR detection of autophagy-associated genes in QBC939 cells (mean ± SD, *n* = 3; ^#^ *p* < 0.05, vs. control and *p < 0.05, vs. cisplatin). **(B)** Western blot detection of autophagy proteins in QBC939 cells. **(C)** Quantitation of p62 and LC3B protein levels (mean ± SD, *n* = 3; *p < 0.05, vs. cisplatin). **(D)** QBC939 cells were treated with cisplatin (10 μg/mL) with or without DCA (20 mM) and CQ (10 μM) for 36 h. Cell viability was determined using MTT assay (mean ± SD, *n* = 3; *p < 0.05, vs. cisplatin and ^#^ *p* < 0.05, vs. cisplatin + DCA).

Then, we examined gene and protein expression levels of autophagy-related molecules using RT-qPCR and western blot. We found that the gene expression levels of ATG5, ATG7, ATG12, and MAP1LC3A were higher in the groups treated with cisplatin combined with DCA than in the control and cisplatin groups. Furthermore, there were no significant increases in these genes’ expression levels in the cisplatin groups compared with the control groups, which was consistent with the PCR array results (Figure 7A). Western blot results showed that the expression of p62 decreased and the expression LC3B increased in the groups treated with cisplatin combined with DCA compared with the control and cisplatin groups, indicating that autophagic flux was activated (Figures 7B, C).

To clarify the role of autophagy induced by DCA in response to cisplatin, we treated QBC939 cells with CQ, an autophagy inhibitor, to detect cell viability using MTT assay. As shown in Figure 7D, the sensitivity of QBC939 cells to cisplatin increased in the groups treated with cisplatin combined with DCA and the groups treated with cisplatin combined with CQ. Moreover, the sensitivity of QBC939 cells to cisplatin significantly increased in the groups treated with cisplatin combined with DCA and CQ. This result demonstrated that inhibition of autophagy can enhance the sensitivity of QBC939 cells to cisplatin under DCA treatment.

## 4 Discussion

Tumor drug resistance is still the main challenge of clinical tumor chemotherapy, as early effective chemotherapy cannot maintain a sustained effective antitumor effect. Previous studies on the mechanism of chemotherapy resistance in tumors have explored molecular signals, organelle interactions, tumor microenvironment, and even population and found that multilevel and multifaceted reasons are involved in the formation of tumor chemotherapy resistance (Chatterjee and Bivona, 2019). Even though an increasing number of new molecular targeted drugs are emerging, these drugs are often only effective for a small number of patients. Thus, it is important to clarify the formation mechanism of tumor chemotherapy resistance, and finding a low-cytotoxicity and high-efficiency combination chemotherapy strategy will provide the potential direction to break the bottleneck of tumor chemotherapy resistance.

Almost a hundred years ago, Otto Warburg first observed the characteristics of abnormal energy metabolism of cancer cells (Warburg et al., 1927); that is, instead of mitochondrial aerobic oxidation under aerobic conditions, cancer cells use glycolysis to provide energy for themselves (Warburg, 1925; Warburg, 1956). This switch of the cellular metabolism model from mitochondrial aerobic oxidation to glycolysis is one of the key hallmarks of cancer cells and is associated with tumor chemotherapy resistance and poor prognosis (Hanahan and Weinberg, 2011). PDH complexes are localized in the mitochondrial matrix and catalyze irreversible decarboxylation of pyruvate to acetyl-CoA and NADH to initiate the tricarboxylic acid (TCA) cycle and oxidative phosphorylation (OXPHOS). PDK contains four subtypes, all of which can inhibit the activity of PDH. Thus, when PDK is inhibited, PDH is activated, and mitochondrial aerobic oxidation is restored (McFate et al., 2008). In tumor cells, upregulated PDK1–4 can inhibit PDH activity and support aerobic glycolysis (Schulze and Downward, 2011; Zhao et al., 2013). Sanmai et al. analyzed the expression of PDK in CCA tissues using proteomics and showed that PDK was highly expressed in CCA tissues (Sanmai et al., 2019). The high expression of PDK in CCA provides evidence for the selection of PDK-specific inhibitor DCA as a chemotherapeutic synergist.

In this study, we used glucose and lactate assay kits to analyze the metabolism of QBC939 cells and RBE cells. The results (Figure 2) showed that DCA increased the level of glucose uptake and reduced the level of lactic acid secretion under cisplatin stress, demonstrating that the cellular metabolism of CCA cells switched from glycolysis to aerobic oxidation. With this metabolic reprogramming, DCA decreased the cell viability of QBC939 cells treated with cisplatin (Figure 1), indicating the important role of glycolysis in cisplatin resistance formation in CCA cells. ROS are a major accessory product of mitochondrial aerobic oxidation, and their pronounced upregulation can activate the intracellular antioxidant system, including superoxide dismutase (SOD), catalase, glutathione peroxidase, and Sirt3 (Ma, 2014; Tong et al., 2015). Bonnet et al. found that DCA could enhance the TCA cycle in tumor cells (A549, M059K, and MCF-7 cells), promote the production of OXPHOS respiratory chain complex I–mediated ROS increase, and reduce cell proliferation (Bonnet et al., 2007). Consistent with this research, our results of intracellular ROS and mtROS levels detected with fluorescence microscopy (Figures 5A, B) showed that DCA increased the levels of intracellular ROS and mtROS in QBC939 cells under cisplatin and activated the expression of the antioxidant system–associated proteins Sirt3 and SOD2. Interestingly, the levels of intracellular ROS and mtROS increased in QBC939 cells treated with cisplatin only, but the expression of SOD2 did not change significantly, suggesting that ROS levels are in a state of stress-priming and that the cell responses can be activated once the extracellular stress is experienced.

Escape from apoptosis and sustained proliferation are the two main hallmarks of tumor cells for chemotherapy resistance (Senga and Grose, 2021). Our apoptosis assay results (Figure 3A) showed that there was no significant increase in apoptosis cell ratio in QBC939 cells treated with cisplatin combined with DCA. Bcl-2 family members can interact with their Bcl-2 homology (BH) domains to play pro- or antiapoptotic functions (Hatok and Racay, 2016; Warren et al., 2019); thus, BCL-2 family members play an integral role in apoptosis. Consistent with the apoptosis assay, RT-qPCR results (Figure 3B) showed that DCA did not upregulate the expression of Bcl-2 family apoptosis-associated genes BAX, BAK, BCL2, BCL2L1, and MCL1 under cisplatin stress. Meanwhile, the expression of apoptosis-associated protein cleaved caspase 3 had no significant changes (Figure 3C), indicating that DCA did not trigger the mitochondria-dependent apoptosis pathway in QBC939 cells. Then, we focused on the cell proliferation and cell cycle. The morphological results (Figure 4A) showed that no fragmented cells (apoptotic cells) were observed in the groups treated with cisplatin only or cisplatin combined with DCA, the total cell count decreased, and the cells became round. The cell population doubling time assay (Figure 4C) showed that DCA significantly increased the cell population doubling time under cisplatin stress in QBC939 cells, indicating the occurrence of cell cycle arrest. As a member of the serine/threonine protein kinase family, cyclin-dependent kinases (CDKs) participate in cell cycle regulation by mediating the phosphorylation of different substrates (Malumbres, 2014; Matthews et al., 2022). The CDK inhibitors (CKIs) mainly interact with cyclin–CDK to prevent cyclin from binding to the corresponding CDK, thereby inhibiting CDK activity and resulting in the stagnation of the cell cycle process (Li et al., 2022). The CKIs include the INK4 family (p16^INK4A^, p15^INK4B^, p18^INK4C^, and p19^INK4D^), CIP/KIP family (p21^Cip1^, p27^Kip1^, and p57^Kip2^), and ribosomal protein-inhibiting CDKs (RPICs) (Bury et al., 2021). Our results of CKI gene expression detected using RT-qPCR (Figure 4B) showed that DCA significantly upregulated the gene expression of CDKN1A (also known as P21CIP1), CDKN2A (also known as P27KIP1), and CDKN1B (also known as P16INK4A) under cisplatin stress. Interesting, cell cycle assay results (Figure 5E) showed that cell cycle arrest induced by cisplatin combined with DCA was significant relieved by inhibiting mtROS using Mito-TEMPO. We assume that these phenomena of nuclear gene overexpression may be caused by mtROS-mediated retrograde signaling from mitochondria to nuclei triggered by DCA, suggesting the potential relationship between DCA-mediated oxidative stress and CCA cells’ cisplatin resistance.

As a response to antagonize oxidative stress, autophagy is necessary to maintain cell metabolism (Gao et al., 2020). In addition to the increase in antioxidant capacity, cells can also increase the degradation of damaged proteins and organelles through the autophagy–lysosome pathway in the face of oxidative stress to maintain cell metabolism and redox homeostasis (Green et al., 2011). Since autophagy is closely related to intracellular antioxidant capacity (Zhang et al., 2009), combined with the above results of the high expression of antioxidant proteins in QBC939 cells treated with cisplatin combined with DCA, a human autophagy RT^2^ Profiler PCR array was used to detect the overall level of intracellular autophagy. As shown in Figure 6, DCA upregulated the majority of autophagy-associated genes expression in QBC939 cells under cisplatin stress compared with the treatment with cisplatin only. Furthermore, RT-qPCR results (Figure 7A) showed that the expression of genes involved in autophagic vacuole formation [ATG5, ATG7, ATG12, and MAP1LC3A (Klionsky et al., 2021)] was upregulated in the groups treated with cisplatin combined with DCA, consistent with the PCR array results. Western blot results (Figures 7B, C) showed that the expression of p62 decreased and the expression LC3B increased in the groups treated with cisplatin combined with DCA compared with those in the control and cisplatin groups, indicating that autophagic flux was activated. Next, cell viability assay results (Figure 7D) showed that the autophagy inhibitor CQ significantly increased the sensitivity of QBC939 cells to cisplatin. These results demonstrated that DCA activated autophagy in QBC939 cells under cisplatin stress, which is a protective machinery for CCA cells to resist cytotoxicity induced by cisplatin combined with DCA.

An increasing number of researchers are investigating the potential mechanisms of tumor metabolic reprogramming in chemotherapy resistance and believe that intervening with or inhibiting the expression of metabolic pathway–related genes holds promise for developing novel antitumor drugs. Chen et al. discovered that hexokinase inhibitor 3-bromopyruvate (3-BrPA) could inhibit energy metabolism and cause the dissociation of Hexokinase II from mitochondria, lead to the release of apoptosis-inducing factor (AIF), and trigger cell death in human leukemia cells (Chen et al., 2016). Furthermore, in the research of human gastric cancer (p-SK4), esophageal cancer (OE33), breast cancer (MCF-7, MDA-MB-468, and MDA-MB-231), and osteosarcoma (U2OS) cells, Cheong et al. observed that the combination of 2DG and metformin was able to induce mitochondrial dysfunction–mediated energy deprivation through impairment, leading to cellular apoptosis (Chen et al., 2016). These results suggest that breaking metabolic reprogramming–mediated cellular homeostasis may be a target to improve the chemotherapy efficacy by multiple pathway intervention.

Collectively, our results provide evidence that DCA changes the metabolic model from glycolysis to aerobic oxidation in CCA cells under cisplatin stress. The metabolic reprogramming leads to mitochondrial redox dysfunction and increases the mtROS level. On the one hand, upregulated ROS as a retrograde signal increase the expression levels of cell cycle arrest genes and antioxidant genes; on the other hand, such high levels of mtROS activate the cell protection machinery of CCA cells, such as autophagy. Inhibition of autophagy further increases the synergistic effect of DCA and cisplatin. These data show that metabolic reprogramming mediated by DCA induces mitochondrial oxidative stress, which increases the sensitivity of CCA cells to cisplatin, and inhibition of mtROS-activated autophagy enhances the sensitization effect of DCA under cisplatin treatment. These results may provide a specific strategy for glycolysis-dependent chemotherapy-resistant tumors and offer evidence for enhancing the clinical synergistic efficacy of multiple agents.

## Data Availability

The original contributions presented in the study are included in the article/Supplementary Material, further inquiries can be directed to the corresponding author.
